# From Disease Silos to Integrated Cardiometabolic Care: Diabetes, Vascular Disease, and Heart Failure

**DOI:** 10.1111/1753-0407.70272

**Published:** 2026-09-21

**Authors:** Zachary T. Bloomgarden, Yehuda Handelsman

**Affiliations:** ^1^ Division of Endocrinology, Diabetes and Bone Disease, Department of Medicine Icahn School of Medicine at Mount Sinai New York New York USA; ^2^ The Metabolic Institute of America Tarzana California USA

## 1 A Meeting Report and Critical Synthesis—Not a Clinical Practice Guideline

**FIGURE 1:**
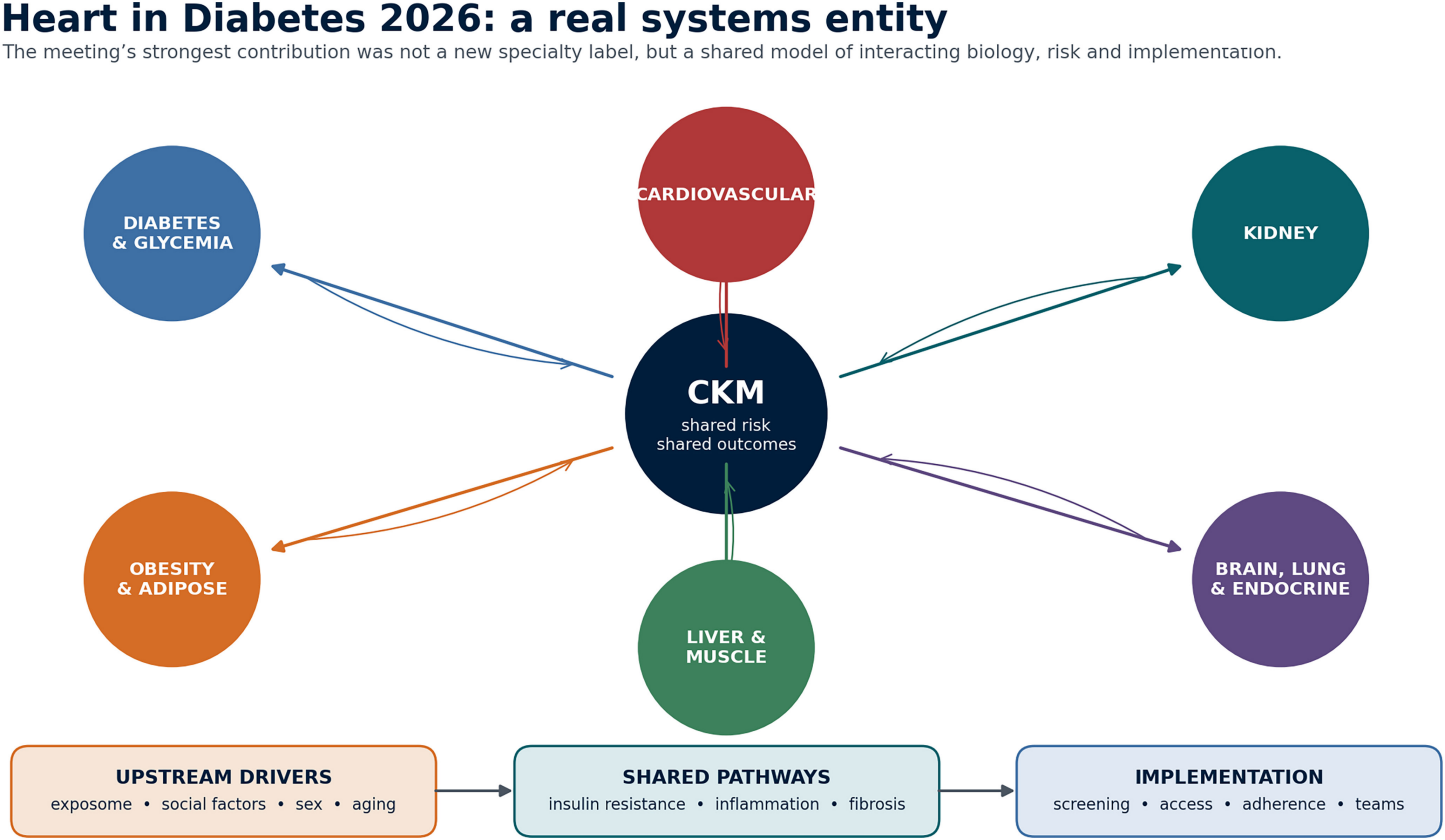
HiD as a systems entity: Shared biology and shared outcomes do not eliminate organ‐specific diagnosis; they require coordinated care.

The Tenth Heart in Diabetes (HiD) meeting, held in Philadelphia, Pennsylvania, June 19–21, 2026, offered a clinical approach to the complex cardio‐renal‐metabolic (CRM) patient. Features of CRM disease include diabetes, insulin resistance, obesity, chronic kidney disease, atherosclerosis, heart failure, fatty liver disease, vascular inflammation, and neurohormonal activation, typically with multiple coexisting conditions which interact biologically and increasingly share treatments. The American Heart Association cardiovascular‐kidney‐metabolic (CKM) framework provides a formal pathophysiologic structure, while HiD emphasizes the practical task of managing these problems together [1, 2] Figure 1.

**FIGURE 2:**
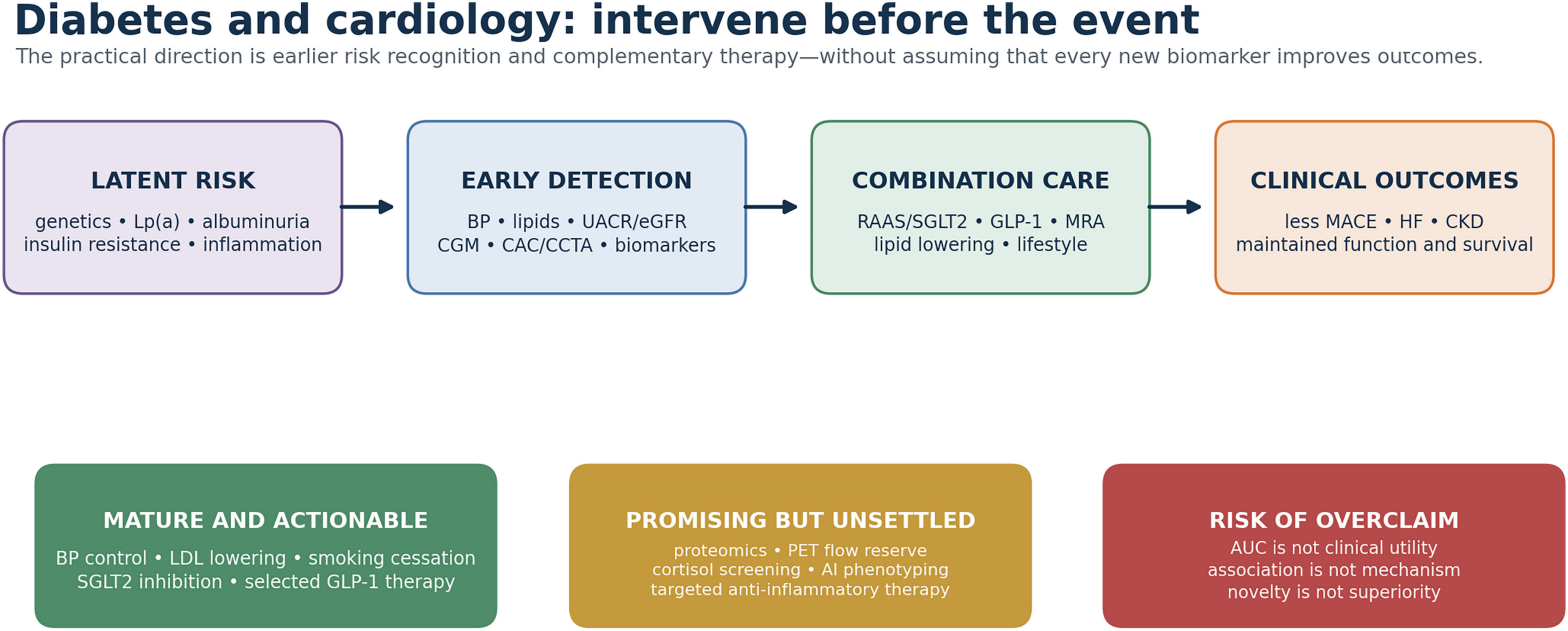
Diabetes and cardiology converge around earlier risk detection and complementary care; the evidence hierarchy must remain visible.

The meeting's strongest message was not that every disorder should be collapsed into a single syndrome. Each patient's risk should be addressed by integrating glucose, lipids, blood pressure, albuminuria, body weight, liver disease, and heart failure instead of treating them as unrelated targets, recognizing that multiple treatment approaches have benefits crossing traditional specialty boundaries. Earlier detection and complementary treatment are justified only when they improve clinical decisions, implementation, and outcomes Figure 2.

## 2 Why the Concepts Underpinning HiD Form a Real Entity

The historical organization of medicine is anatomical: the heart, cardiology, the kidney, nephrology, glucose, and endocrinology. The biological organization of disease is different. Insulin resistance, adipose dysfunction, endothelial injury, inflammation, fibrosis, sympathetic activation, aldosterone signaling, impaired natriuresis, and social disadvantage do not respect departmental boundaries. Albuminuria predicts cardiovascular events; heart failure risk rises as kidney function falls; obesity drives diabetes, metabolic‐associated steatotic liver disease (MASLD), atrial fibrillation, and heart failure with preserved ejection fraction (HFpEF); and therapies first developed for glucose lowering now improve kidney, heart failure, liver, and atherosclerotic outcomes. The AHA definition of CKM syndrome—pathophysiologic interactions among metabolic risk factors, CKD, and the cardiovascular system leading to multiorgan dysfunction and adverse cardiovascular outcomes—captures this reality [1, 2].

HiD is therefore more than a meeting title. Its conceptual legitimacy rests on three observations. First, the same patients repeatedly cross specialty boundaries. Second, common pathways generate correlated organ injury. Third, the strongest therapies have become cross‐organ interventions. SGLT2 inhibitors, GLP‐1 receptor agonists, nonsteroidal MRAs, intensive lipid lowering, and effective obesity treatment cannot be assigned neatly to one organ. The field is moving from “Which specialist owns this disease?” toward “Which combination of mechanisms, tests, and implementation supports this patient's risk trajectory?”

CKM/CRM should not become a vague container into which every chronic condition is placed. A unifying framework is useful only if it improves detection, treatment selection, accountability, and outcomes. It is not a substitute for diagnosing the cause of kidney disease, distinguishing heart failure with reduced ejection fraction (HFrEF) from HFpEF, identifying familial lipid disorders, recognizing endocrine hypertension, or determining whether obesity has produced objective organ dysfunction. The danger of a broad systems label is that it can create conceptual coherence while concealing diagnostic heterogeneity. HiD then represents a care model and risk network, not necessarily as one uniform disease. Its value will be judged by whether it reduces fragmentation, therapeutic delay and preventable events—not by how many organs can be added to the acronym.

## 3 Diabetes and Glycemic Phenotyping

### 3.1 Type 1 Diabetes Belongs Within CKM Thinking

The meeting appropriately resisted restricting CKM disease to T2D. Adults with Type 1 diabetes (T1D) increasingly live with overweight or obesity, hypertension, dyslipidemia, kidney disease, and insulin resistance. Peripheral insulin delivery creates a nonphysiologic portal‐peripheral gradient, and the insulin dose needed to overcome resistance can further promote weight gain. The clinically important point is not whether this should be called “double diabetes,” but that cardiovascular and kidney risk in T1D cannot be inferred from A1c alone.

Adjunctive incretin therapy in T1D remains promising rather than routine. Meta‐analyses and observational experience suggest reductions in body weight, insulin dose, and postprandial exposure, but ketosis, hypoglycemia, gastrointestinal effects, and insulin‐adjustment errors remain potential concerns. The 2026 Phase 2 trial of tirzepatide in adults with T1D and obesity supports efficacy, but it is not sufficient to establish broad use or long‐term cardiorenal benefit [3]. The meeting was strongest when it framed the question as phenotype‐directed adjunctive therapy, not as a general replacement for careful insulin management.

### 3.2 Beyond A1c: CGM, Diet and Heterogeneity

CGM‐derived time in range, time below range, and glycemic variability provide clinically meaningful information that A1c cannot. An unresolved issue is whether each CGM metric independently predicts cardiovascular outcomes or whether much of the apparent association reflects mean glucose and disease severity. The meeting did not establish that time in range should be treated as a cardiovascular surrogate. It is better viewed as a patient‐centered glycemic measure with strong relevance to treatment safety and daily burden.

The notion of DASH4D (Dietary Approaches to Stop Hypertension for Diabetes), a modified version of the classic DASH diet, reinforces a less glamorous but durable principle: Diet quality remains crucial and is not an optional background recommendation. A DASH‐style pattern adapted to T2D can improve blood pressure and glycemic variability, and its effect does not depend on a novel receptor target. New drugs should complement, not displace, nutrition, sleep, smoking cessation, and physical activity.

### 3.3 Combination Therapy: Additive Mechanisms, Incomplete Direct Testing

The rationale for combining an SGLT2 inhibitor and a GLP‐1 receptor agonist is compelling: The classes differ in glycemic, weight, hemodynamic, heart‐failure, kidney, and atherosclerotic effects. However, “complementary” should not automatically be translated as “synergistic.” Most outcome evidence comes from trials of one class added to background care, subgroup analyses, or meta‐analytic comparisons—not from large factorial trials designed to quantify incremental benefit for every endpoint. Combination use is often reasonable, but the expected benefit should be linked to the individual patient's residual risk rather than to a generalized desire to assemble every pillar.

### 3.4 Endocrine Phenotypes That Masquerade as Treatment Resistance

The aldosterone and cortisol presentations challenged the reflex diagnosis of “resistant hypertension” and “resistant diabetes” (“difficult to treat diabetes”) as nonadherence or therapeutic failure. Primary aldosteronism should be considered in resistant hypertension, and hypercortisolism can present without classic Cushingoid appearance in a substantial subset of patients with disproportionate degrees of hyperglycemia, visceral adiposity, fractures, thrombosis, atrial fibrillation, or heart failure. Mifepristone improved glycemia in the CATALYST treatment phase among selected patients with T2D and hypercortisolism [4]. Targeted case‐finding rather than broad indiscriminate screening is appropriate.

## 4 Cardiology, Vascular Disease and Heart Failure

### 4.1 Atherosclerosis Before the Conventional Threshold

The ASCVD sessions emphasized that major events occur in people who do not meet traditional “very high risk” definitions. Short‐ to medium‐term calculators may underestimate lifetime risk. Lifetime exposure, polygenic susceptibility, lipoprotein(a), albuminuria, inflammation, and early plaque should be seen as substantial risk markers in individuals with apparently modest elevations in LDL‐C or blood pressure. This argues for earlier and more aggressive risk factor reduction when the total pattern is convincing, particularly in diabetes and CKD. It does not mean that every patient requires advanced imaging or maximal pharmacotherapy.

Coronary CT angiography (CCTA) can reveal noncalcified plaque and earlier atherosclerosis that a coronary artery calcium (CAC) score or functional test may miss. The value is greatest when the result will change treatment or clarify symptoms. Imaging without a decision pathway, however, risks converting uncertainty into incidental findings, repeated testing, and anxiety. Likewise, proteomic scores may improve discrimination, but a statistically better risk model has little clinical value unless it reclassifies patients accurately and leads to treatment that improves outcomes.

### 4.2 Lipid Disorders: Earlier, Deeper and More Individualized

The lipid sessions supported earlier LDL‐C lowering, use of ApoB when residual atherogenic particle burden is uncertain, one‐time or targeted Lp(a) measurement, and CAC or CCTA when treatment decisions remain genuinely uncertain. Rare disorders such as homozygous familial hypercholesterolemia and familial chylomicronemia are increasingly treatable through PCSK9, ANGPTL3, and ApoC‐III pathways. The conceptual advance is important: Not all high triglycerides or high LDL‐C represent the same biology.

The new therapies should still be judged against old ones. Statins, ezetimibe, blood‐pressure control, and smoking cessation remain inexpensive, outcome‐proven, and underused. An oral PCSK9 agent, a gene‐silencing therapy, or base editing may transform adherence or durability, but novelty, though promising, still requires long‐term comparative safety and effectiveness assessment, and the difficulty of patient access must be considered. The meeting presented exciting potential updates to our therapeutic pipeline, but the development of realistic approaches to implementation must inform clinical recommendations.

### 4.3 Acute Coronary Syndromes and Post‐MI Therapy

The ACS update highlighted plaque rupture versus erosion, radial access, intracoronary imaging, antithrombotic strategy, and inflammatory risk. The nuanced discussion of beta‐blockers was particularly useful: The historical routine continuation after myocardial infarction may not confer the same benefit in a revascularized patient with preserved ejection fraction and no other indication as in the patient with HFrEF or ventricular dysfunction. This is an example of progress through subtraction—recognizing when older treatments are no longer automatically appropriate.

### 4.4 Hypertension: The Largest Implementation Failure

Blood‐pressure treatment exhibits a major gap between evidence and practice. Only lately have experts agreed on lower goals of < 130/80 and even < 120/80 for higher risk patients with diabetes, CKD, and stroke history. More accurate blood pressure measurement, therapeutic inertia, nonadherence, cost, fragmented follow‐up, and failure to use combination pills often matter more than lack of pharmacologic options. Dietitian‐delivered medical nutrition therapy reduces blood pressure and improves related risk markers [5]. Blood‐pressure lowering also belongs within brain‐health protection, and is not only important for stroke and heart‐failure prevention [6].

Novel agents—angiotensinogen silencing, aldosterone synthase inhibition, and endothelin antagonism—may help selected patients. Before these are adopted, however, they must be shown to outperform or meaningfully complement inexpensive existing therapy. Aldosterone synthase inhibitors could theoretically avoid “aldosterone escape” and non‐genomic aldosterone effects, but although it is now recognized that low BP improves outcome regardless of the agents used, these new agents need to demonstrate benefit and safety. For the present time they should be described as promising, not superior. Aprocitentan and endothelin‐pathway drugs have anti‐proteinuric and nocturnal BP effects but remain constrained by edema, volume retention and heart‐failure risk.

### 4.5 Heart Failure, Microvasculature and the Broader Cardiovascular Phenotype

The heart‐failure sessions reinforced that clinically “stable” patients remain at high absolute risk. NT‐proBNP distributions rise as eGFR falls, but renal dysfunction does not invalidate natriuretic peptide measurement, which continues to convey prognostic information. HFpEF does not have a single etiology: Obesity, diabetes, CKD, atrial fibrillation, pulmonary vascular disease, and systemic inflammation produce overlapping phenotypes. The favorable effects of GLP‐1 RA and GLP‐1/GIP RA in obesity‐related HFpEF, with and without Type 2 diabetes, support targeting the metabolic phenotype, but they do not establish equivalent benefit in all HFpEF patients. Their impact on HFrEF has not been proven yet, and studies are ongoing [7, 8].

A session at the meeting of positron emission tomography (PET) imaging proposed myocardial flow reserve as a window into microvascular CKM disease and linked skeletal muscle quality and intramuscular fat to myocardial perfusion. This is conceptually attractive, but it is uncertain whether measuring flow reserve in asymptomatic CKM patients changes management beyond intensive risk‐factor treatment. The STRIDE trial provides a more direct example of functional outcome improvement: Semaglutide improved walking capacity in symptomatic peripheral artery disease and T2D [9].

The proposal that the lung may be a diabetes‐related microvascular target is biologically plausible and clinically useful when unexplained dyspnea persists. Restrictive physiology, impaired diffusion, pulmonary vascular abnormalities, and reduced exercise capacity should not automatically be attributed to deconditioning. However, routine pulmonary screening in asymptomatic diabetes is not established, and pulmonary hypertension remains etiologically heterogeneous.

### 4.6 Inflammation as a Treatment Target

The 2026 Luminary Lecture and Award for the Heart in Diabetes (HiD) conference was presented to Peter Libby, who delivered a lecture entitled “Inflammation in Cardiometabolic Disease: No longer a theory.” The NLRP3 Inflammasome complex, interleukin (IL)‐1, IL‐6, C‐reactive protein (CRP), clonal hematopoiesis, and myelopoiesis should all be viewed within a causal model of atherothrombosis. The challenge is not proving that inflammation matters; it is finding interventions and when to intervene for a favorable net clinical effect. Colchicine and IL‐1 pathway inhibitor studies demonstrate that inflammatory risk can be modified, but infection, tolerability, and patient selection limit the broad use of this approach. The observation that weight loss, incretin therapy, or metabolic surgery lowers CRP does not by itself prove that inflammation mediates their cardiovascular benefit.

## 5 Critical Appraisal

**Table:** 

Established/actionable	Emerging/plausible	Needs skepticism
Risk assessment beyond A1c; routine use of CGM for insulin‐treated diabetes; aggressive management of BP, lipids, and kidney risk.	Phenotype‐directed adjunctive therapy in Type 1 diabetes; combination SGLT2/GLP‐1 treatment; endocrine case‐finding.	Treating CGM metrics as validated CV surrogates; assuming all combination effects are additive; broad cortisol screening without phenotype selection.
BP control, LDL‐C/ApoB lowering, smoking cessation, HF guideline‐directed therapy, indicated SGLT2 use, and selected GLP‐1 therapy.	CCTA in selected uncertainty, PET flow reserve, anti‐inflammatory selection, Lp(a)‐specific therapy, and endocrine hypertension pathways.	Screening by advanced tests without action thresholds; assuming a new lipid or BP drug is better than inexpensive established therapy; conflating biomarker change with outcome benefit.

The practical implication is that integration should sharpen, not blur, clinical reasoning. The next commentary in this series turns to kidney disease, obesity, muscle, and liver, where the same tension exists: Powerful cross‐organ therapies are changing care, but surrogate findings and mechanistic plausibility must not be mistaken for improved health.

## Funding

The authors have nothing to report.

## Conflicts of Interest

The authors declare no conflicts of interest.
